# Progressive ataxia, cognitive decline, urinary incontinence, and unexplained hydrocephalus: a rare case of idiopathic normal pressure hydrocephalus in epileptic patient

**DOI:** 10.1097/MS9.0000000000002959

**Published:** 2025-01-31

**Authors:** Raju Shah, Tek Nath Yogi, B.C Pooja, Amrit Bhusal, Ashesh Koirala, Shailendra Katwal, Shishir Shahi, Rijan Kafle, Ranjan Shah

**Affiliations:** a BP Koirala Institute of Health Sciences (BPKIHS), Dharan, Koshi, Nepal; b Department of Psychiatry; BP Koirala Institute of Health Sciences (BPKIHS), Dharan, Koshi, Nepal; c Department of Radiology; Tribhuvan University Institute of Medicine, Kathmandu, Nepal; d Manipal Pokhara College of Medical Science, Pokhara, Kaski, Nepal

**Keywords:** cognitive impairment, gait disturbance, idiopathic normal pressure hydrocephalus, lumbar puncture, neuroimaging, ventriculoperitoneal shunt

## Abstract

**Introduction:**

Idiopathic normal pressure hydrocephalus (iNPH) poses diagnostic challenges due to its overlapping symptoms with other neurodegenerative disorders. The clinical trial of gait disturbance, cognitive impairment, and urinary incontinence often mimics other conditions, making accurate diagnosis crucial.

**Case presentation:**

A 67-year-old male with a 40-year history of epilepsy presented with progressive gait abnormalities, cognitive decline, and bladder incontinence over 5 years. Initial evaluation revealed a magnetic gait, impaired short-term memory, and a Mini-Mental State Examination score of 17/30. MRI and CT scans showed ventriculomegaly without elevated cerebrospinal fluid (CSF) pressure. A lumbar puncture with a positive CSF tap test confirmed iNPH, and ventriculoperitoneal shunt surgery was performed, resulting in significant improvement in symptoms.

**Discussion:**

iNPH is characterized by the triad of gait disturbance, cognitive impairment, and urinary incontinence. Its diagnosis is primarily based on clinical presentation and neuroimaging. The CSF tap test is a crucial diagnostic tool, and shunt surgery remains the standard treatment. This case underscores the need for a high index of suspicion for iNPH in patients with atypical presentations and emphasizes the importance of long-term follow-up to monitor treatment efficacy.

**Conclusion:**

This case highlights the complexity of diagnosing iNPH, particularly in patients with comorbid conditions such as epilepsy. Thorough diagnostic evaluations and timely intervention are critical for improving patient outcomes. Long-term monitoring is essential to assess the effectiveness of treatment and manage potential complications.

## Introduction

**HIGHLIGHTS**

- Idiopathic normal pressure hydrocephalus (iNPH) is a complicated diagnosis, especially when it comes to distinguishing it from other neurodegenerative illnesses because of overlapping symptoms, as the manuscript skillfully demonstrates. Accurate diagnosis is crucial, as evidenced by the well-documented comprehensive diagnostic evaluation that includes neuroimaging and the CSF tap test.
- The case involves a 67-year-old male with a long history of epilepsy, presenting with progressive gait abnormalities, cognitive decline, and bladder incontinence over 5 years. His Mini-Mental State Examination score was notably low at 17/30, indicating significant cognitive impairment.
- MRI and CT scans revealed ventriculomegaly without elevated cerebrospinal fluid (CSF) pressure, which is characteristic of idiopathic normal pressure hydrocephalus (iNPH). This imaging was crucial for the diagnosis.
- A lumbar puncture was performed, yielding a positive CSF tap test, which confirmed the diagnosis of iNPH. This procedure not only aided in diagnosis but also had therapeutic implications.
- Following the diagnosis, the patient underwent ventriculoperitoneal shunt surgery, which resulted in significant improvement in his symptoms, highlighting the effectiveness of surgical intervention in iNPH cases.
- The case underscores the necessity for long-term follow-up to evaluate treatment efficacy and manage potential complications, emphasizing the complexity of diagnosing and treating neurological disorders like iNPH.

Normal intraventricular pressures are a defining feature of normal pressure hydrocephalus (NPH), a type of communicating hydrocephalus. Urinary incontinence, cognitive decline, and gait impairment are the three symptoms that first appear. When the cause of a condition is unknown, it is referred to as idiopathic normal pressure hydrocephalus, or iNPH^[1]^. The prevalence studies’ results showed that crude overall rates ranged from 10/100 000 to 22/100 000 for probable iNPH and 29/100 000 for potential iNPH. Age-specific rates, on the other hand, varied from 3.3/100 000 for those in the 50–59 age range to 5.9% for those who were 80 years or older. The overall crude rates from incidence studies ranged from 1.8/100 000 to 7.3/100 000 per year, while age-specific rates varied from 0.07/100 000/year in individuals under 60 years of age to 1.2/1000/year in individuals over 70 years of age^[2]^. iNPH is estimated to affect approximately 6% of the elderly population, yet it remains underdiagnosed due to overlapping symptoms with other neurodegenerative diseases^[3]^. Cerebrospinal fluid (CSF) dynamics, or abnormal CSF generation, kinetics, and reabsorption, are the cause of intractable paroxysmal paralysis (iNPH). Furthermore, vascular, metabolic-neurodegenerative, and genetic variables are involved^[4]^. When they attempt to walk, people with NPH have a unique gait that has been compared to being magnetic. It looks as though their feet are cemented to the ground.^[5]^ NPH dementia is a subcortical dementia marked by poor executive function and forgetfulness^[6]^. Idiopathic normal-pressure hydrocephalus must be diagnosed by neuroimaging using either CT or MRI. Although it is not specific to iNPH, an Evans ratio of more than 0.3 implies large ventricles, and a ratio of more than 0.33 indicates very large ventricles^[7]^. While ventriculoatrial shunts were more frequently used in the past, ventriculoperitoneal (VP) shunts are now the standard surgical treatment for infarcted posterolateral hemolysis (iNPH)^[1]^. The terms “intractable paroxysmal paralysis” and “infarcted posterolateral hemolysis” refer to distinct medical conditions. The former is associated with periodic paralysis, a rare neuromuscular disorder characterized by episodic attacks of muscle weakness due to ion channel mutations, often triggered by factors like diet or exercise^[8,9]^. The latter, while not explicitly defined in the provided literature, suggests a condition involving hemolysis in the context of infarction, potentially linked to myoglobinuria, where muscle damage leads to myoglobin release and subsequent hemolytic processes^[10]^.

This case has been reported in line with SCARE guideline^[11]^.

## Case presentation

A 67-year-old Asian male patient initially appeared with a 5-year history of progressively declining gait abnormalities, forward fall tendency, bladder incontinence, and forgetfulness of recent occurrences and one and a half years history of hypertension. He walked with a very sluggish, unsteady, ataxic, magnetic gait with little arm swing. Step height is significantly reduced, creating the impression that the person’s feet are immobile. His concentration and short-term memory were both compromised, yet he was able to speak without any difficulty. His thinking seemed to be slower and more forgetful. Nonetheless, he could complete basic things on his own. He was diagnosed with epilepsy 40 years ago after experiencing a seizure that caused him to lose consciousness and was prescribed medicine. His last seizure had occurred 9 years prior to his presentation to our clinic.

The patient was conscious and well oriented to time, place and person. The patient’s vital signs were as follows: blood pressure of 130/80 mmHg, oxygen saturation of 96% as determined by a pulse oximeter in room air, pulse rate of 74 beats per minute, body temperature of 98°F, and respiration rate of 20 breaths per minute. Pallor, icterus, lymphadenopathy, cyanosis, edema, or dehydration were not present. Upon examination of the lower limbs, normal sensitivity, coordination, reflexes, tone, and power were found. Rhomberg’s test result was positive, he had trouble doing the alternate hand movement and was unable to perform the finger nose test. On arrival, his Mini-Mental State Examination was 17/30. Initial laboratory studies (Table 1) revealed neutrophilia, elevated AST, ALT and triglycerides.

**Table 1:** Laboratory investigations of patient

Test	Result	References
Hemoglobin (mg/dL)	14.7	13–18
PCV (%)	45.6	45–50
TLC (cells/mm3)	10 700	4000–11 000
DLC (%)	N 71 L 23	N40-60 L20-40
Platelets (cells/mm3)	3 24 000	1 50 000–5 00 000
MCH (pg)	26.1	27–32
MCHC (g/dL)	29.4	30–35
MCV (fL)	98.7	76–96
Serum urea (mg/dL)	31	13–43
Serum creatinine (mg/dL)	0.84	0.6–1.2
Serum Sodium (meq/L)	141	135–155
Serum potassium (meq/L)	4.6	3.5–5.5
Liver function test		
Total protein (g/dL)	7.8	6–8.3
Total bilirubin (mg/dL)	0.98	0.3–1.2
Conjugated bilirubin (mg/dL)	0.21	<0.4
ALT (U/L)	41.4	05–34
AST (U/L)	36.1	05–31
ALP (U/L)	139.4	64–306
Triglyceride (mg/dl)	168	40–150

ALP, alkaline phosphatase; ALT, alanine transaminase; AST, aspartate transaminase; DLC, differential leucocyte count; Hb, hemoglobin; MCH, mean corpuscular hemoglobin; MCHC, mean corpuscular hemoglobin concentration; MCV, mean corpuscular volume; TLC, total leucocyte count; WBC, White blood cells.

**Figure 1:**
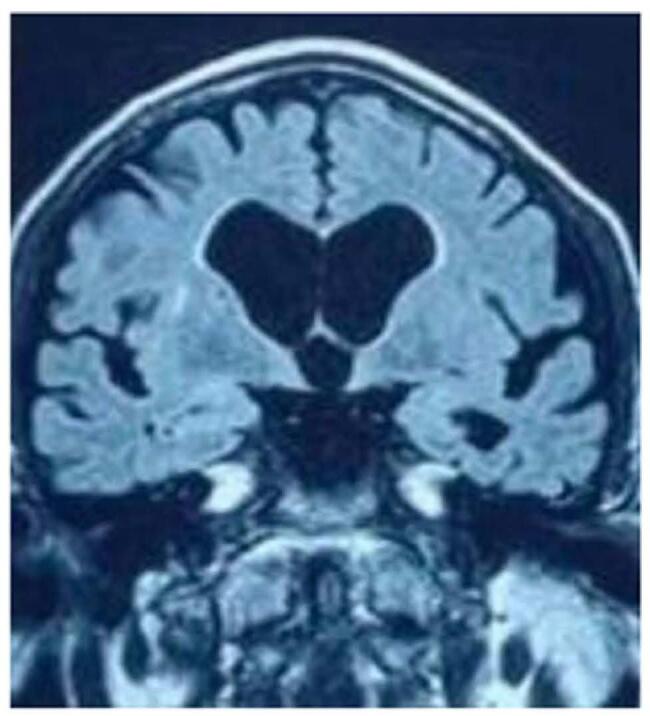
Axial T2 weighted MRI image at the level of basal ganglia showing the prominence of the sylvian fissure as compared to other sulci. Dilated ventricles also noted.

**Figure 2:**
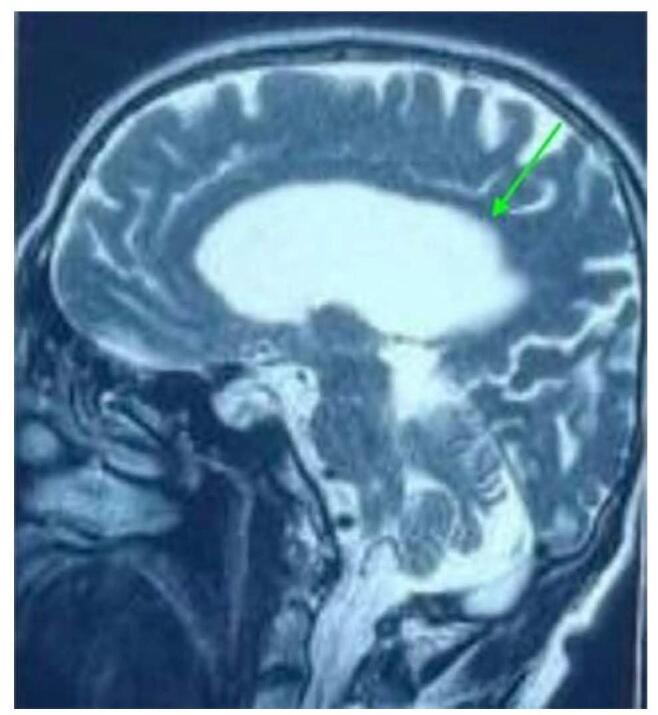
T2 weighted mid sagittal MRI image showing the posterosuperior bowing of the corpus callosum (green arrow) with prominent ventricles.

**Figure 3:**
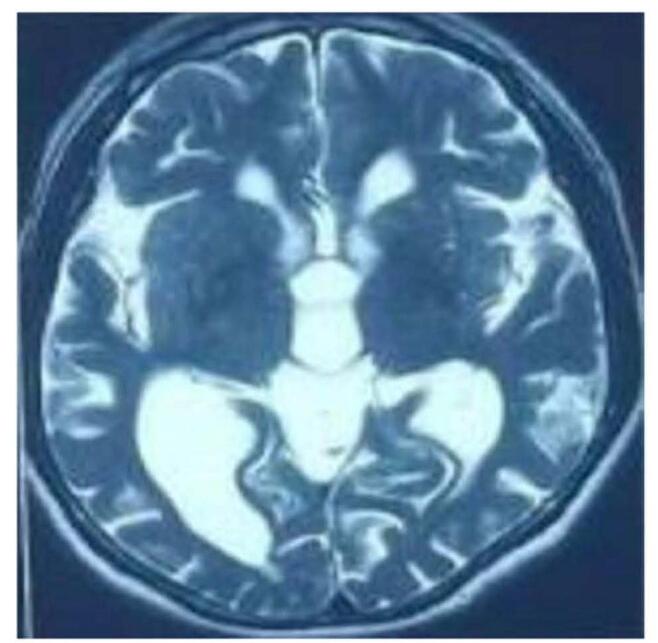
T1 weighted coronal MRI image showing the prominent of bilateral temporal horn of lateral ventricle without features of hippocampal atrophy.

In MRI, Fig. 1 reveals axial T2 weighted MRI image at the level of basal ganglia showing the prominence of the sylvian fissure as compared to other sulci. Dilated ventricles also noted. Fig. 2 shows T2 weighted mid sagittal MRI image showing the posterosuperior bowing of the corpus callosum (green arrow) with prominent ventricles. Fig. 3 reveals T1 weighted coronal MRI image showing the prominent of bilateral temporal horn of lateral ventricle without features of hippocampal atrophy. CT scan revealed dilated ventricles. In view of above clinical features with Hakim’s Triad consisting of gait disturbance, dementia and urinary incontinence and the results of the neuroimaging and CSF tap test, primary working diagnosis of NPH was made and we admitted the patient. 50 mL of CSF was extracted after lumbar puncture. The following day, the gait improved as shown in Supplementary Digital Content, Video, and the MMSE increased from 17 to 20. CSF Dynamic test was not available. Patient was planned for reevaluation after 3 months.

## Discussion

Idiopathic NPH and secondary NPH are the two major subtypes of NPH. The latter can result from subarachnoid hemorrhage and intraventricular hemorrhage brought on by trauma or aneurysms, meningitis-related diseases, inflammatory disorders, or other uncommon causes such Paget’s disease of the skull^[12]^. The three main manifestations of NPH are urine incontinence, cognitive disturbance, and trouble with gait^[13]^. These symptoms are thought to result from dysfunction of the periventricular white matter tracts and supplementary motor areas of the frontal cortex, especially those that provide connections to the frontal lobe^[14]^. Different descriptions of the INPH gait pattern exist, such as “magnetic,” “glue-footed,” or “apractic^[15]^.” Having trouble walking at the desired pace, ascending stairs and stopping walking on an incline are the most frequent concerns^[15]^. The majority of the cognitive deficits usually seen in INPH patients are subcortical in nature and include slowed information processing, deviations in mood without any evidence of localized brain impairments, psychomotor slowness, memory and executive function abnormalities, and visuospatial deficits^[16-18]^. Initially, there is more often an increase in frequency and/or urgency of urination; as INPH worsens, frank urine incontinence develops^[14]^. Parkinson’s disease (PD), vascular dementia, Alzheimer’s disease (AD), and aids dementia complex are among the differential diagnoses for INPH^[19]^.

“Frontal-subcortical” dysfunction is the hallmark pattern of NPH, characterized by a patient’s poor learning and better retained recognition memory, trouble with fluency tests, poor performance on divided attention and executive function tests, and slower completion of timed tasks^[17,20]^. INPH is diagnosed based on clinical presentation, radiographic findings, and supplemental prognostic tests. Radiographic diagnosis of INPH is based on the identification of ventriculomegaly in the absence of elevated CSF pressure. This finding can be observed through imaging techniques such as MRI or CT scans^[21]^. In order to temporarily alleviate symptoms, a tiny volume of CSF is extracted during the CSF tap test, a diagnostic technique. Following the tap test, improvements in gait and cognitive function point to a confirmed diagnosis of NPH^[22]^. CSF dynamics testing, including continuous monitoring of CSF pressure, has been explored but showed limited correlation with symptomatic improvement. Despite its potential, the complexity and variability in results may explain its exclusion in some cases, as it does not consistently predict shunt responsiveness^[23]^. A parametric model for MRI mean diffusivity histogram analysis can be a helpful tool in differentiating NPH from other neurodegenerative diseases including PD and AD^[24]^. Given its improved imaging capabilities over CT, MRI is thought to be the ideal modality for diagnosing NPH. Improved anatomical conditions assessment, white matter alteration detection, and flow-void sign evaluation are all possible with MRI^[25]^. Given that the clinical symptoms of NPH can mimic those of other neurodegenerative conditions, neurophysiological and neuropsychological methods can aid in the clinical evaluation of NPH patients and aid in the differential diagnosis of NPH and dementia^[26]^. In a study, individuals who had relief from their symptoms after a high-volume tap responded well to shunting at three months and reported benefits at 3 years^[27]^.

VP shunt implantation is the most frequently performed surgical method for the management of iNPH. In retrospective research, this was the situation for 40 of the 42 patients, and major improvements in brain MRI, cognitive function, and urinary symptoms were noted^[1]^. Patients who react to CSF drainage or whose CSF hydrodynamic factors are consistent with iNPH may consider shunt surgery^[7]^.

- However there are some potential post-operative complications with this surgery, most commonly infections and blockages. Studies show that infection rates in VPS patients might reach 37.9%, which raises serious concerns about postoperative infections^[28]^. Obstruction of the distal end of VPS tubes is common, with laparoscopic interventions proving effective in resolving these issues in 77.5% of cases^[29]^. Other complications may include intracerebral or intraventricular hemorrhage, malposition, abdominal perforation during placement, shunt erosion of the skin with exposure of the system, shunt over drainage (slit ventricles), shunt nephritis, subdural hematomas, abdominal CSF collections (pseudocyst or CSFoma)^[30]^.

The case report provides detailed information about the patient’s clinical presentation, symptoms, and diagnostic findings, allowing for a comprehensive understanding of the case. The report includes the results of imaging studies, such as MRI and CT scans, which help in the diagnosis of NPH. The case report highlights the positive response to lumbar puncture and subsequent improvement in gait and cognitive function, which supports the diagnosis of NPH. The integration of additional diagnostic tools, particularly Lumbar Infusion Study (LIS) is a valuable diagnostic tool for assessing CSF dynamics in INPH patients, offering insights beyond the tap test with lower morbidity^[31]^. However due to the weaker financial status of the patient they could not afford for the test.

The duration of follow-up and long-term outcomes of the patient are not mentioned, making it difficult to assess the effectiveness of the treatment or the progression of the condition. The case report does not discuss potential confounding factors or alternative explanations for the observed symptoms, which may limit the interpretation of the findings.

Hydrocephalus is not commonly recognized as a cause of seizures in general, although epilepsy is reported to be frequently associated with shunt-treated hydrocephalus, especially in children^[32]^. A total of 32% of the children were diagnosed with epilepsy, often with onset coinciding with the hydrocephalus diagnosis. Most of the kids who were impacted had severe, uncontrollable seizures. The initial source of the hydrocephalus has a major impact on the incidence of epilepsy^[33]^. In the study conducted by Laresson *et al*, it was found that 4.5% people with NPH have epilepsy. The rates of seizures before and after shunt implantation have been examined in certain studies. Research indicates that the insertion of a shunt may be linked to epilepsy; however, this could be because people with more severe hydrocephalus have a higher chance of having epilepsy as well as being shunted^[33]^.

Seizures may be observed in patients with hydrocephalus after a subarachnoid hemorrhage or post-traumatic hydrocephalus and also as complication following shunt procedures to relieve intracerebral hematomas, subdural effusions, or hematomas. Seizures have been reported as a postoperative complication in patients with iNPH^[34,35]^. Long-term epilepsy can be a factor in the development of iNPH^[36]^. Like the above studies, our case having the long history of epilepsy had developed iNPH manifesting epilepsy to be potential risk factor for iNPH. So the diagnostic evaluation and timely intervention play a crucial role in the patient’s symptomatic relief, and quality of life.

## Conclusion

The case study emphasizes the value of thorough diagnostic examinations, including neuroimaging and lumbar puncture, and emphasizes the relevance of taking into account iNPH in individuals with unique symptoms. The lumbar puncture’s strong reaction highlights its potential for both diagnosis and treatment. The report does, however, stress the importance of long-term monitoring including the use of appropriate patient-reported outcome measures (PROMs) to evaluate the effectiveness of treatment. The case study underscores the importance of maintaining high suspicion for iNPH, especially in patients with atypical symptoms or comorbidities. Proper education, clinical experience, and thorough diagnostic evaluations, including neuroimaging and lumbar puncture, are essential for accurate diagnosis. Long-term monitoring ensures treatment efficacy and manages complications. All in all, it highlights how intricate neurological disorders may be and how crucial a precise diagnosis is for illnesses like iNPH.

## Data Availability

Not applicable.
